# Actively incorporating lifestyle modifications into daily life: The key to adherence in a lifestyle intervention programme for metabolic syndrome

**DOI:** 10.3389/fpubh.2022.929043

**Published:** 2022-08-01

**Authors:** Qun Wang, Sek Ying Chair, Eliza Mi Ling Wong, Xichenhui Qiu

**Affiliations:** ^1^School of Nursing, Shenzhen University, Shenzhen, China; ^2^The Nethersole School of Nursing, The Chinese University of Hong Kong, Shatin, Hong Kong SAR, China; ^3^School of Nursing, Tung Wah College, Kowloon, Hong Kong SAR, China

**Keywords:** adherence, lifestyle intervention, metabolic syndrome, qualitative research, experiences

## Abstract

**Introduction:**

Lifestyle modifications are the first-line interventions for metabolic syndrome (MetS) management. The effectiveness of lifestyle interventions depends mostly on participants' adherence to the interventions. The current study was to explore the experiences of MetS patients in attending lifestyle intervention program (LIP) and the factors that influenced their adherence to the interventions.

**Methods:**

A descriptive qualitative study was designed following the COREQ guideline. Face-to-face semi-structured individual interviews were conducted with a purposive sample from the participants who attended the LIP using the data saturation principle. Content analysis of transcripts was conducted following the methods proposed by Graneheim and Lundman.

**Results:**

The study recruited 27 participants, including 13 males and 14 females. Four themes were identified: (i) the positive and beneficial experiences of attending the LIP, including incorporating lifestyle modifications into daily life, improved physical and psychological health, and empowerment; (ii) facilitators of adherence, including individualized lifestyle education, regular follow-ups, and adequate interpersonal support; (iii) barriers to adherence, including personal resistance, competing demands, and contextual factors; (iv) suggestions for future interventions: with multidisciplinary team, longer term intervention, and more efficient approaches. The findings also indicated that young-to-middle aged patients faced more conflicts with role-related commitments, and were open for e-approaches in lifestyle interventions.

**Conclusion:**

The LIP provided positive and beneficial experiences for the participants. Actively incorporating lifestyle modifications into daily life is the key to maintain participants' adherence to the LIP. Culturally appropriate and psycho-behavioral strategies should be adopted to overcome personal and contextual barriers. Special attentions should be paid for the young-to-middle aged population in MetS management.

## Introduction

Metabolic syndrome (MetS) is a cluster of cardio-metabolic risk factors, including central obesity, elevated blood pressure, hyperglycaemia and dyslipidaemia (1). MetS has become prevalent because of the global rise of sedentary lifestyles and unhealthy diet. It was estimated that about 25.0% of the global population had MetS (2). In China, the prevalence of MetS increased from 8.8% in 1991–1995 to 29.3% in 2011–2015 (3). Under the interactions of multiple risk factors, people with MetS have increased risks for cardiovascular diseases, diabetes, and stroke (4). Hence, the prevention and management of MetS have become a global public health concern.

International guidelines consistently recommended the healthy lifestyle, including regular exercise, healthy diet, smoking cessation and effective stress management, as the first-line treatment for MetS (1, 5). As the professional with the most frequent involvement in lifestyle interventions, nurses play critical roles in MetS management (6–8). The nurse-led lifestyle intervention programme (LIP) has been widely applied in MetS management. Previous nurse-led LIPs usually contain the education, counseling, and telephone follow-ups with different formats. For example, one nurse-led 12-week LIP employed one counseling session with a brochure, and weekly telephone-based motivational interviewing sessions to promote the physical activity levels among middle-aged and older women with MetS (9). In another study, the nurse provided a low-intensity interventions for MetS management, including two 90-minute group-based education sessions, one 15-minute individual counseling session, and two telephone follow-ups (10). A recent study provided booklet-based or app-based education together with a nurse-led face-to-face education session for adults with MetS (7). These studies revealed significant improvements in waist circumference, systolic blood pressure and the quality of life (7, 9, 10). Most nurse-led LIPs required home-based behavior modifications, such as healthy diet, regular exercise with specific goals like 30-min brisk walking per day, or 8,000–1,000 steps per day. Among the nurse-led LIPs which had various interventional components and delivery formats, participants' experiences are important to understand the mechanism and key components of interventions (11, 12). However, evidence of participants' experiences in attending the nurse-led LIP for MetS management is lacking.

As reported elsewhere, we developed a 3-month nurse-led LIP for Chinese adults with MetS (13, 14). A registered nurse provided a 10-chapeter lifestyle modification booklet, one face-to-face health education session (about 30 mins) and six biweekly telephone follow-ups (about 20 mins) to the participants in the intervention group. Underpinned by the Health Promotion Model (HPM), the LIP employed strategies to improve health promoting behaviors, such as goal setting and empowerment. A randomized controlled trial (RCT) was conducted to examine the effectiveness of this LIP quantitatively, and revealed significant improvements in body weight, self-efficacy, health promoting behaviors, depression, and quality of life. Although with improving trends, no significant differences were detected in some physical indicators, such as waist circumference, lipid files, and glucose (13). Changes in the physical indicators depended mostly on participants' implementation of and adherence to the interventions (15). The HPM also emphasized participants' commitment to a plan of an action, which could lead to the implementation of behaviors (16). Understanding the facilitators of and barriers to participants' adherence to interventions is necessary to better interpret quantitative findings (11). Due to the limitation of the quantitative design, the RCT did not provide evidence on participants' adherence or the influencing factors. Qualitative study methods could provide rich and comprehensive information about values, beliefs and cultural influences on behaviors. Rashidi et al. (15) reviewed the published qualitative studies and synthesized the influencing factors of patients' adherence to cardiovascular disease treatment plans. They identified both internal and external factor that influenced patients' adherence, including perceptions and beliefs on lifestyle modification, exercise and taking medication; and the extent of support and mentorship. However, there is limited study on the influencing factors of adherence to the LIP for MetS.

Moreover, participants' experiences of attending the LIP will also provide valuable clues for further improvements of the interventions. Based on the qualitative findings on patients' perceptions of nurse-led self-management interventions for chronic obstructive pulmonary disease, insights for improvements were summarized (17). Jones et al. (18) collected participants' experiences of attending community-based mindful walking in the pilot study. Their findings indicated the feasibility and acceptability of the intervention, and provided directions for improvement in the main study. Torres-Vigil et al. (19) employed a qualitative study to explore the role of nursing calls with advanced cancer patients in oncology care, which identified the key elements of effective interventions. However, evidence of experiences in nurse-led interventions for MetS management is lacking. The current study aimed to describe the participants' experiences of attending the nurse-led LIP for MetS management, and to explore the facilitators of and barriers to their adherence to the intervention.

## Methods

### Design

The study employed a descriptive qualitative study design and used content analysis of transcripts. Semi-structured face-to-face individual interviews were conducted after the completion of the RCT. The current study report followed the Consolidated Criteria for Reporting Qualitative Research (20).

### Participants

The participants were recruited from patients who received the nurse-led LIP in the RCT (n = 76). The inclusion and exclusion criteria of patients with MetS for the RCT were reported elsewhere (14). In summary, the participants were Chinese adults aged over 18 years and with MetS following the IDF definition. People who had psychiatric illnesses or terminal diseases (e.g., cancer and heart failure), with difficulties in taking moderate-intensity physical activity (such as osteoporosis and osteoarthritis), or could not communicate in Chinese were excluded. Purposive sampling was used to recruit participants with diverse age, gender, occupation and educational background. The sample size was determined by the information saturation principle. For content analysis, a sample size of 15–20 is required (21). In the current study, three more interviews were conducted when data saturation was detected to ensure that no new information emerged.

### Data collection

The study followed the principles outlined in the Declaration of Helsinki. Ethical approval was obtained from the ethics committee of the university (No. CRE-2014.068). Written consent was obtained from each participant before the start of the interview.

Potential participants were invited by telephone calls. The study aims and procedures were explained in the invitation calls. Voluntary participation was assured. Appropriate time was appointed with the participants who agreed to attend the study. Data was collected by WQ, a female PhD candidate when conducting the study and with training in qualitative research. The participants knew the interviewer, as she also administered the consent procedures in the RCT. The interviews were conducted in a quiet room of the study hospital, where only the interviewer and the participant were presented. The participants were interviewed once. All interviews were audio-recorded, and field notes were made by the interviewer. The interviews lasted for 30–45 mins.

A semi-structured interview guide was developed based on the literature to address the study objectives (11, 22, 23). SYC and EMLW reviewed and edited the guide. The three main questions are as follows. (1) What were your experiences during your participation in the nurse-led LIP? (2) What factors influenced your adherence to the LIP? (3) What are your suggestions for improvement? Follow-up and probe questions were asked to collect detailed information about patients' feelings, perceived benefits, and the facilitators and barriers to adherence to the LIP. Three pilot interviews were conducted and no change was made in the interview guide. Therefore, the pilots were included in the final data analysis.

### Data analysis

All interviews were conducted in Chinese, and the researchers' mother language is Chinese; therefore, data analyses were conducted in Chinese. The audio records were transcribed verbatim immediately after the interviews. Transcriptions were checked manually for accuracy by XCHQ. Two researchers (WQ and EMW) read the transcripts and conducted content analysis independently following the methods proposed by Graneheim and Lundman (24), Table 1. The two researchers' analyses were compared and the differences were discussed until a consensus was achieved. A third researcher (SYC) reviewed all the findings on codes, sub-themes and themes. After data analysis, WQ translated all the findings (themes, sub-themes, codes and citations) into English. The English and Chinese versions of the study findings were compared by SYC and XCHQ to ensure their equivalence.

**Table 1:** Content analysis steps.

**Steps**	**Analysis**
1	Reading the transcripts for several times to obtain an overall understanding of the phenomenon
2	Labeling the meaningful units with words, sentences or paragraphs, and condensing similar meaningful units into codes
3	Sorting the codes into sub-themes according to their similarities and differences.
4	Formulating the sub-themes into main themes by their meanings and content

### Trustworthiness

Following the rigor evaluation principles proposed by Lincoln and Guba (25), several strategies were employed to ensure the credibility, transferability, dependability and confirmability of the present study. Firstly, all interviews were administered using an interview guide, which ensured that the data were relevant to the studied phenomenon. Secondly, member checking was performed to ensure credibility. By the end of each interview, the researcher confirmed the main points with the participants and sought for feedback or corrections. The analysis findings were sent to three participants to check whether the interpretations were consistent with their experiences (26). Thirdly, all codes, sub-themes and themes were cross-checked among the research team to establish the confirmability. Fourthly, all interviews were audio-taped, and detailed field notes were made. The rich and detailed documentation established the transferability of the study. Fifthly, all the researchers are experienced in qualitative research and content analysis. As the researchers were familiar with the original RCT, they may hold some preconceptions on the study phenomenon. Therefore, bracketing was practiced by the researchers to prevent the impacts of their perceptions of the LIP on understanding the participants' experiences. The bracketing approach would help the researchers keep reflexivity and neutrality in data collection and analysis, demonstrating the validity of the study (27). As introduced in the Data Analysis section, independent data analysis, comparisons and discussions were conducted until consensus was achieved. This process also established the confirmability of this study. All authors are fluent in English and Chinese, which ensured the equivalence of the translated analysis findings.

## Results

Twenty-seven participants were interviewed and five patients refused to attend the interview because they had limited time or were visiting their children in another city. The characteristics of the participants are listed in Table 2.

**Table 2:** Characteristics of the participants (*n* = 27).

**Characteristics**	**Groups**	***n*** **(%)**
Age (range: 33–73)	≤ 50	6 (22.22)
(Mean: 57.19 ± 10.19)	50–60	11 (40.74)
	>60	10 (37.04)
Gender	Males	13 (48.15)
	Females	14 (51.85)
Occupation	Retired	14 (51.85)
	White collars	5 (18.52)
	Blue collars	3 (11.11)
	Farmers	5 (18.52)
Education years	≤ 9 years	6 (22.22)
	9–12 years	13 (48.15)
	>12 years	8 (29.63)
Medical history	Hypertension	21 (77.78)
	Diabetes	17 (62.96)
	Dyslipideamia	21 (77.78)
Smoking history	Current smoker	3 (11.11)
	Quit smoking	4 (14.81)
	Never smoked	20 (74.08)

**Figure 1:**
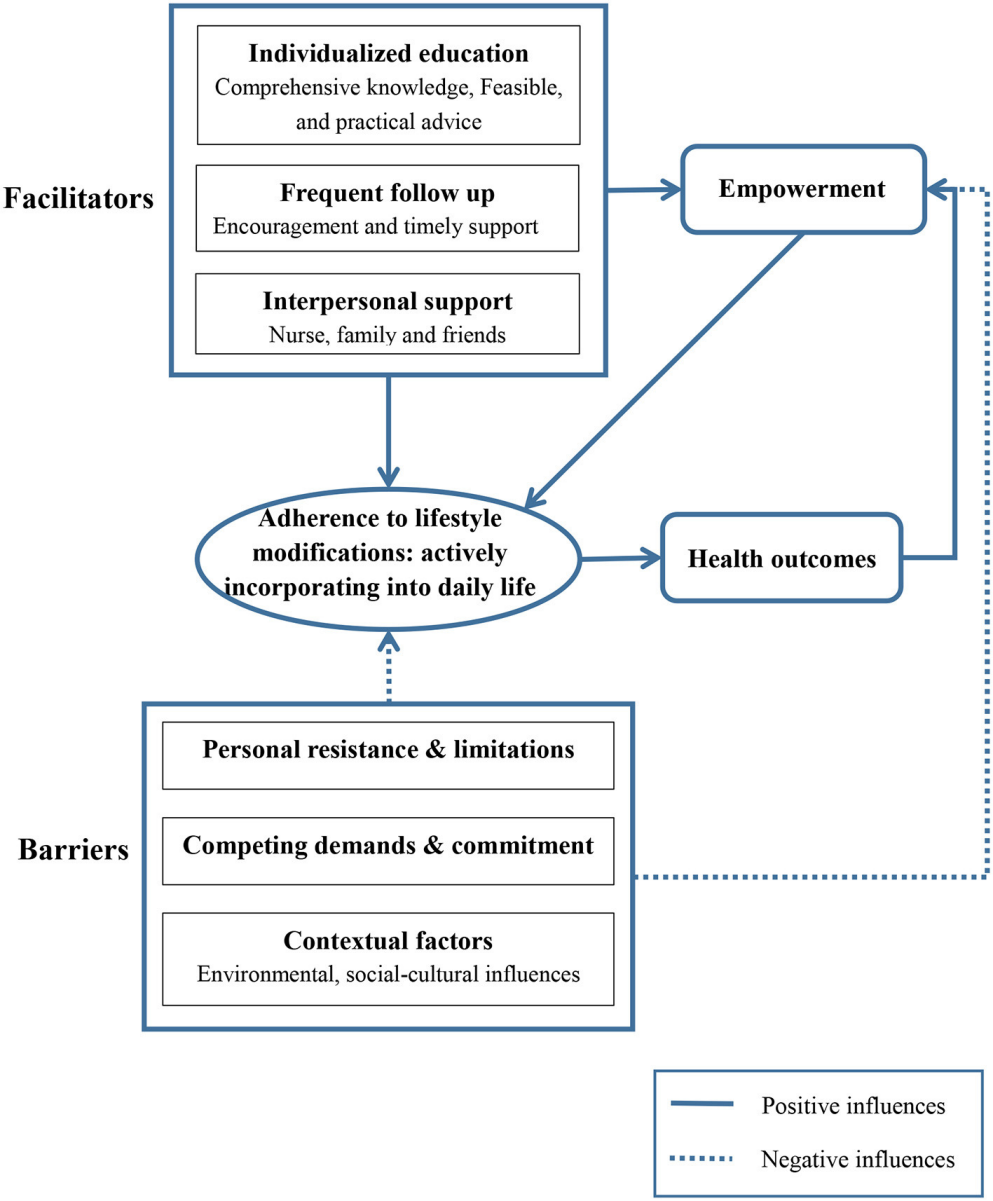
The influences of different factors on adherence to lifestyle interventions.

The data analysis revealed four themes: positive and beneficial experiences of attending the LIP, facilitators of adherence to the LIP, barriers to adherence to the LIP, and suggestions for further improvement (Table 3). The influences of different factors on adherence to lifestyle modifications are presented in Figure 1.

**Table 3:** Qualitative findings of transcribed interviews (*n* = 27).

**Themes**	**Sub-themes**
The positive and beneficial experiences in attending the lifestyle intervention programme	• Actively incorporating lifestyle modifications into daily life
	• Improved physical and psychological health
	• Empowerment for health management
Facilitators of adherence to the lifestyle intervention programme	• Individualized lifestyle education
	• Regular follow-ups
	• Adequate interpersonal support
Barriers to adherence to the lifestyle intervention programme	• Personal resistances and limitations
	• Competing demands and commitment
	• Contextual factors
Suggestions for improvements	• Multidisciplinary team
	• Longer-term intervention
	• More efficient intervention approach

### The positive and beneficial experiences of attending the LIP

The participants provided positive comments and appreciations for the nurse-led LIP. During the programme, they incorporated the lifestyle modifications into daily life, perceived different benefits led by the behavior changes.

#### Actively incorporating lifestyle modifications into daily life

Most participants mentioned that they changed the lifestyles after attending the LIP. Some had actively incorporated exercise and healthy diet into their daily life. For example, some patients climbed stairs instead of using elevators, kept walking or standing during entertainment, and took more food with high-fiber and low-calorie. With acceptable attitudes and active incorporation, the participants regarded the healthy lifestyle as “*a part of the life*” and were more likely to sustain the behavior changes.

*My diet and exercise habits have changed remarkably. Now, walking has become a part of the life. I walk to the markets and the factory instead of driving. You know, I would drive for only 100 meters in the past. Now I keep walking when watching TV*. (Participant 09, male, 50 years)

*It feels comfortable to have regular exercise and light diet. If I had more food in one meal, I would reduce the intake in the next meal. If I missed exercise one day, I would do more in the next day... I usually walk at night. Last Friday, one of my old friends visited me. We had dinner together and talked till 10 pm. That night, I did eat a bit more food than usual, and not walk. In the next day, I walked for a longer time and had light diet*. (Participant 15, male, 60 years).

#### Improved physical and psychological health

The patients perceived various benefits after attending the LIP, including improved physical and psychological health, and empowerment for health management. Some participants reported the feelings of “lightness,” “full of energy,” “less fatigue or pain” with weight loss.

*Now, look, my belly gets smaller in these months. Bending is much easier. My body turns to be sturdy and strong. I am full of energy again*. (Participant 01, male, 71 years).

*I was too fatigue to go upstairs before. Now after losing some weight, I could go to the 4th floor easily. I keep doing housework. No more pains or strains*. (Participant 04, female, 61 years).

*I was diagnosed with non-alcoholic fatty liver disease in previous body check. Now it disappeared! This is another benefit of lifestyle changes*. (Participant 14, male, 33 years).

Some participants mentioned improvements in psychological health, with “positive,” “relaxed,” and “peaceful” minds. Someone also perceived better sleep quality.

*Now, with more exercise in the day time, my mood is more peaceful. I can fall asleep faster and sleep tighter. My mood is much better now*. (Participant 07, female, 59 years).

#### Empowerment for health management

With the successful behavior changes and improved health, some participants mentioned they rebuilt confidence for health management.

*I tried to lose weight many times, but none succeeded. This time, I achieved the goal in 3 months. This inspires me a lot. Now, I am confident to handle my health problems*. (Participant 19, female, 50 years).

### Facilitators of adherence to the LIP

The participants mentioned that the sessions of education, follow-ups and interpersonal support as the facilitators of adherence to the LIP, which helped them incorporate lifestyle interventions into daily life.

#### Individualized lifestyle education

Every participant provided positive comments to the LIP. They specifically appreciated the “comprehensive” and “individualized” education, and the “feasible” and “practical” advice, which facilitated the incorporation of healthy lifestyles into daily life.

*This education is only for me and tailor-made for my conditions. I learned diet, exercise, mental health, smoking and drinking, medication, and monitoring. Very comprehensive. It also considered my personal preferences. I could follow it in daily livings*. (Participant 07, female, 59 years).

*The nurse taught me practical methods to measure the quantity, such as one cola-bottle cap of soy sauce, one beer-bottle cap of salt and two bowls of vegetables. It is very clear and easy to use*. (Participant 18, female, 60 years).

#### Regular follow-ups

Most participants valued the biweekly telephone follow-ups. During the calls, the nurse “reminded,” “encouraged,” and “motivated” the participants to follow the interventions, and provided timely feedback to their progress, which facilitated better adherence to lifestyle modifications.

*She (the nurse) reminded me to keep the (lifestyle) changes. I was motivated by her encouragements and praise. I would not keep the habits without the follow-up*. (Participant 04, female, 61 years).

#### Adequate interpersonal support

Interpersonal supports from the nurse, family members and friends were regarded as facilitators of adherence to the LIP. The nurse led the LIP and provided professional support. Most participants mentioned that the feelings of “warm,” “happy,” and “safe” when being cared by the nurse. These positive feelings facilitated their adherence to the interventions.

*The nurse is very nice. I'm happy for being cared. I am moved by her professional spirits and caring attitudes during the education and calls. This inspired me to stay in the programme*. (Participant 13, female, 57 years).

The family and friends accompanied and supported the participants to keep healthy lifestyles in daily life.

*My mom and wife helped me a lot. They prepared healthy meals and encouraged me to exercise every day. Without their help, I could not stick with it*. (Participant 14, male, 33 years).

*My friends invited me to square dance. Sometimes I wanted to quit. They insisted and called me. With their accompany, I danced every night*. (Participant 23, female, 68 years).

### Barriers to adherence to the LIP

The participants also met difficulties in following the lifestyle modifications, including personal resistance and limitations, competing demands and commitment, and contextual factors.

#### Personal resistances and limitations

Some participants talked about their preferences for over-eating, fatty or salty food, or sedentary lifestyles. When taking the light food, they felt “*sad,” “unsatisfied,” “not happy.”* The personal resistances hindered their adherence to the LIP.

*I started to take pickles since childhood. I know it is not good for the health. I am used to eating it everyday. If I had one meal without pickles, it felt like I missed one meal* (Participant 22, male, 54 years).

Some participants experienced pain or other physical morbidity that impeded their adherence to exercise.

*My back pain relapsed last month. It hurt even taking a few steps. I had to quit exercise for a period*. (Participant 16, female, 67 years).

#### Competing demands and commitment

The participants also mentioned the role-related competing demands and commitment that conflicted with lifestyle modifications, such as taking care of the family and work commitment.

*My grandson was in fever last week. My husband and I stayed at home to take care of him. Then, we did no exercise*. (Participant 26, female, 69 years).

*I must take the business trip. I had to drive and stay in the car for days, with instant food and no exercise. It is hard for me to keep the healthy lifestyles*. (Informant 27, male, 39 years).

#### Contextual factors

Some environmental and social-cultural factors were mentioned as barriers for lifestyle modifications. The bad weather and lack of facilities were sometimes stated as the reasons for quitting exercise.

*It is summer and so hot. Biking would cause a lot of sweat. It feels uncomfortable. So I quit riding*. (Participant 11, female, 52 years).

*I used to walk in the stadium at night, but it closed for maintenance. I stopped walking since then*. (Participant 20, male, 40 years).

Some cultural beliefs and customs were mentioned as barriers for their lifestyle modifications. For example, someone held wrong beliefs about obesity.

*People at my age all had a hard time in childhood. The food supply was limited. So, we think obesity is a good thing. The one who is obese must have a good living. If anyone obtains weight recently, we would eat more food to catch up with him*. (Participant 02, male, 52 years).

Various banquets for celebrations, receptions and friend-gathering are being held in local life. Drinking more wine and eating more food are believed to indicate better interpersonal relationship. Therefore, attending the banquets was mentioned as a barrier for lifestyle modification, especially for the male patients who were at work and active in social activities.

*People in Shandong are hospitable. I have to attend some receptions and business meals. Most food there are high in calories and fat. White wine is inevitable. I also had to take second-hand smoking. You know, face is very important for us. It is impolite if I do not drink with the guests*. (Participant 17, male, 55 years).

### Suggestions for future lifestyle interventions

The participants also provided suggestions for improvement in future lifestyle interventions: inclusion of a multidisciplinary team, longer-term intervention, and using more efficient delivery approach.

#### Multidisciplinary team

Although the LIP was developed by professionals from different disciplines, only the research nurse contacted with the participants during education and follow-ups. Six participants suggested the inclusion of other healthcare professionals, like physicians and pharmacists.

*I also take many medications for diabetes and hypertension. The team would be perfect if it also has physicians and pharmacists*. (Participant 03, male, 61 years).

#### Longer-term intervention

The current intervention lasted for 3 months. Eighteen participants expressed their willingness of attending the LIP, especially the follow-ups, for a longer term.

*If the nurse could call me for a longer time, I would achieve greater progress*. (Participant 19, female, 50 years).

#### More efficient intervention approach

Some younger participants suggested e-approaches in delivering lifestyle interventions, like e-mail, QQ (an instant chat software) and video-chat. The characteristics of high-efficiency and low-cost in e-approaches were recommended.

*I use computer and smart phones. We can contact with QQ. You can send me messages with pictures, articles and reminders. We can use video-chat for follow-up. It's efficient and without cost*. (Participant 14, male, 33 years).

## Discussion

To the best of our knowledge, this was the first qualitative study that explored participants' experiences of attending the nurse-led LIP for MetS management. The participants had positive and beneficial experiences in the 3-month LIP. The findings revealed different factors that influenced the adherence to the LIP. Actively incorporating the lifestyle modifications into daily life was the key to facilitate participants' adherence. Areas for improvements were also suggested.

The qualitative findings confirmed the feasibility, acceptability, and effectiveness of the nurse-led LIP in MetS management. The participants had beneficial experiences in attending the LIP: completing behavior changes, achieving health improvements, and obtaining empowerment, which were in line with our quantitative findings (13, 14). Participants' active incorporation of the LIP into daily life was the key for their long-term adherence, and played essential roles in achieving the physical, psychological and behavioral improvements. Although following the requirements of the LIP means adherence, the participants had different perceptions and feelings about this experience. In the beginning of the LIP, some participants did followed the interventions, such as daily walking to improve physical activity levels, taking low-fat and low-sodium food. But they met strong internal resistances, as their adherence brought “unhappy” or “no satisfaction” (e.g., participant no. 20 and 22). Once meeting any conflicts or resistance, like weather changes, they quit. These passive lifestyle changes would not last longer. On the other hand, some participants followed the interventions with acceptable attitudes (e.g., participant no. 9 and 15). The active following of the interventions could brought feelings of happiness and fulfillment. The positive feelings and perceptions on behavioral changes and improved health outcomes in turn empowered them for further lifestyle modifications and improvements. This positive circle (Figure 1) echoed the conceptual structure of self-efficacy in the HPM (16) and revealed the remarkable role of psycho-behavioral strategies in lifestyle interventions (15, 17). Some participants reported improved sleep quality, which was not included in the RCT outcomes and could be examined in future studies.

As indicated by the participants, factors that helped incorporate the LIP into daily life (such as individualized education, feasible and practical lifestyle advice) facilitated their adherence. The individualized education helps to build the knowledge base about MetS and lifestyle modifications, and to identify the special significance of lifestyle modifications to themselves. The follow-up and interpersonal support facilitated the participants to keep healthy lifestyles in long term. Therefore, the lifestyle recommended by the LIP changed into “a part of the life.” The current findings also indicated the successful characteristics of the LIP (15, 19). The individualized, feasible and practical characteristics made the participants feel being specially cared (28), and facilitated the incorporation of lifestyle modifications into daily life. The nurse-led LIP was perceived as providing professional support with a caring attitude, which added evidence to the acceptability and effectiveness of nurse-led interventions. Consistent with previous findings, the caring and empathy components of nurse-led interventions were especially appreciated (17, 19).

Personal, external, and role-related factors were identified as barriers to adherence, where efforts and strategies should be made in future LIPs. The barriers of personal resistances, weather and facilities were commonly reported (15, 17, 28), which indicated the necessity for psycho-behavioral strategies in MetS management. Following the HPM, setting achievable goals and fitting for participants' expectancy would increase participants' self-efficacy to overcome the barriers (16). Group-based interventions, such as peer-supported exercise, would motivate participants to fight against personal resistances (29; Baker and Fatoye, 2018).

The study also indicated social-cultural influences on adherence to the LIP. Firstly, the famine experience shaped some wrong health beliefs amongst local people, such as “obesity means a good living.” Given that the prevalence of MetS increases with age, most of our participants aged over 50 years, who had experienced the Chinese Famine (1959–1961) in their early life. Previous studies reported adverse impacts of the famine experience on people's health beliefs and lifestyle modifications, such as preferences to fatty food and over-eating, leading to increased risks for cardio-metabolic diseases (29, 30). Our qualitative results were consistent with the previous findings. Secondly, local dietary preferences for salty and fatty food impeded adherence to healthy dietary patterns. The study was conducted in Shandong, with a long cold winter every year. Local people make salty pickles to reserve vegetables in winter. Fatty food, which could provide more energy against the cold weather, is also cherished. People have been used to this kind of diet since childhood; therefore, dietary changes are difficult to make in this population (28). Thirdly, the banquet and wine culture conflicted with healthy lifestyles. Under the atmosphere of “hospitality,” people hold rich banquets and drink lots of white wine, which conflicted with their adherence to lifestyle modifications. This finding indicated that lifestyle interventions must be culturally appropriate (31). Public health education should be provided to correct the wrong ideas about weight and promote healthy lifestyles, such as taking low-salt and low-fat food. Instead of banquets and drinking wine, other gathering activities should be advocated, such as group-based physical activities and drinking tea (32). Moreover, the LIP could make full use of the beneficial components in local culture, such as the emphasis on responsibility for family and work, to motivate people to take lifestyle changes.

Our findings suggest that culturally appropriate and psycho-behavioral strategies should be designed to overcome the barriers. As suggested by the participants, a multidisciplinary team could be included, such as lectures and consultations led by physicians and pharmacists. The participants mentioned that a longer-term LIP, especially follow-up calls, would generate more improvements. This might explain the non-significant changes in some physical outcomes in the 3-month RCT. A LIP with a longer intervention period could be designed if adequate support is available.

The current study also indicated that special attention should be paid to the young-to-middle aged people with MetS. With younger age, this group of patients will benefit most from lifestyle modifications. However, their family and work commitments often induce conflicts with lifestyle interventions. Therefore, special strategies should be provided to deal with these conflicts, such as tips for doing office-based exercise and choosing healthy food in the banquets. Moreover, the young-to-middle aged people were open for e-approaches in health management, such as mobile phone applications and online programme. Future studies should fully explore the needs of this population and design specific programme for them.

The current study contributed to a better understanding of participants' experiences of attending a nurse-led LIP for MetS. There are also some limitations. Firstly, we only explored the experiences of the participants. The perspectives of intervention providers and other stakeholders are also valuable (12). Future studies could interview the research nurse and healthcare professionals to understand the perceptions of implementing the LIP and how to incorporate the LIP into routine MetS care. Secondly, the interviewer had known the participants before the interviews. This fact facilitated to build a trust relationship and participants' free expressions in the interviews. All interviews were conducted by WQ, who was the principle investigator of the RCT and had knew the participants before the interviews. The researchers were all familiar with the original RCT. Although the researchers practiced bracketing before the study, it may induce some bias and assumptions in data collection and analysis. Future studies could employ an independent interviewer to prevent this kind of impacts. Thirdly, as all participants were recruited from one hospital in Shandong, cautions should be made when generalizing the findings to other populations.

## Conclusion

In conclusion, the participants had positive and beneficial experiences in attending the LIP. Actively incorporating lifestyle modifications into daily life is the key to maintain participants' adherence to the LIP. Culturally appropriate and psycho-behavioral strategies should be adopted to overcome barriers to adherence to lifestyle interventions. Future LIPs could design longer-term interventions with multidisciplinary professionals and more efficient approaches. Special attention should be paid for the young-to-middle aged MetS patients and to overcome the cultural barriers in lifestyle interventions.

## Data availability statement

The raw data supporting the conclusions of this article will be made available by the authors, without undue reservation.

## Ethics statement

The studies involving human participants were reviewed and approved by Joint Chinese University of Hong Kong—New Territories East Cluster Clinical Research Ethics Committee (No. CRE-2014.068). The patients/participants provided their written informed consent to participate in this study.

## Author contributions

QW wrote the manuscript. SC, EW, and XQ revised the manuscript. All authors made substantial contributions to conception and design, acquisition of data, and analysis and interpretation of data.

## Funding

The study was supported by Top Ranking Projects of Shenzhen University (Nos. 86000000210 and 860000002110118) and Starting Fund of Top-Level Talents of Shenzhen (No. 000513).

## Conflict of interest

The authors declare that the research was conducted in the absence of any commercial or financial relationships that could be construed as a potential conflict of interest.

## Publisher's note

All claims expressed in this article are solely those of the authors and do not necessarily represent those of their affiliated organizations, or those of the publisher, the editors and the reviewers. Any product that may be evaluated in this article, or claim that may be made by its manufacturer, is not guaranteed or endorsed by the publisher.

## References

[1] International Diabetes Federation. The IDF Consensus Worldwide Definition of the Metabolic Syndrome. Belgium: IDF (2006).

[2] Saklayen MG. The global epidemic of the metabolic syndrome. Curr Hyperten Rep. (2018) 20:12. 10.1007/s11906-018-0812-z 29480368 PMC5866840

[3] Huang JJ Huang JL Withers M Chien KL Trihandini I Elcarte E. Prevalence of metabolic syndrome in Chinese women and men: a systematic review and meta-analysis of data from 734,511 individuals. Lancet. (2018) 392:S14. 10.1016/S0140-6736(18)32643-6

[4] Mottillo S Filion KB Genest J Joseph L Pilote L Poirier P. The metabolic syndrome and cardiovascular risk a systematic review and meta-analysis. J Am Coll Cardiol. (2010) 56:1113–32. 10.1016/j.jacc.2010.05.034 20863953

[5] Alberti KG Eckel RH Grundy SM Zimmet PZ Cleeman JI Donato KA. Harmonizing the metabolic syndrome: a joint interim statement of the International Diabetes Federation Task Force on Epidemiology and Prevention; National Heart, Lung, and Blood Institute; American Heart Association; World Heart Federation; International Atherosclerosis Society; and International Association for the Study of Obesity. Circulation. (2009) 120:1640–5. 10.1161/CIRCULATIONAHA.109.192644 19805654

[6] Bassi N Karagodin I Wang S Vassallo P Priyanath A Massaro E. Lifestyle modification for metabolic syndrome: a systematic review. Am J Med. (2014) 127:1242. 10.1016/j.amjmed.2014.06.035 25004456

[7] Wong EML Leung DY Tam HL Wang Q Yeung KW Leung AYM. The effect of a lifestyle intervention program using a mobile application for adults with metabolic syndrome, versus the effect of a program using a booklet: a pilot randomized controlled trial. Clinic Intervent Aging. (2021) 16:633–44. 10.2147/CIA.S303920 33888981 PMC8057802

[8] Marcos-Delgado A Hernández-Segura N Fernández-Villa T Molina AJ Martín V. The effect of lifestyle intervention on health-related quality of life in adults with metabolic syndrome: a meta-analysis. Int J Environ Res Public Health. (2021) 18:887. 10.3390/ijerph18030887 33498570 PMC7908372

[9] Lin CH Chiang SL Heitkemper MM Hung YJ Lee MS Tzeng WC. Effects of telephone-based motivational interviewing in lifestyle modification program on reducing metabolic risks in middle-aged and older women with metabolic syndrome: a randomized controlled trial. Int J Nurs Stud. (2016) 60:12–23. 10.1016/j.ijnurstu.2016.03.003 27297365

[10] Lo SWS Chair SY Lee IFK. Effects of lifestyle intervention on physiological outcomes in Chinese adults with, or at high risk of, metabolic syndrome. J Cardiovascul Nurs. (2017) 32:514–21. 10.1097/JCN.0000000000000386 28060083

[11] Ellard D Parsons S. Process evaluation: understanding how and why interventions work, In: Thorogood M Coombes Y editors. Evaluating Health Promotion: Practice and Methods (3rd Eds). Oxford: Oxford University Press. (2010).

[12] Saarijärvi M Wallin L Bratt E-L. Process evaluation of complex cardiovascular interventions: how to interpret the results of my trial? Euro J Cardiovascul Nurs. (2020) 19:269–74. 10.1177/1474515120906561 32054300 PMC7065447

[13] Wang Q Chair SY Wong EML. The effects of a lifestyle intervention program on physical outcomes, depression, and quality of life in adults with metabolic syndrome: a randomized clinical trial. Int J Cardiol. (2016) 230:461–7. 10.1016/j.ijcard.2016.12.084 28040281

[14] Zheng XJ Yu HB Qiu XCH Chair SY Wong EML Wang Q. The effects of a nurse-led lifestyle intervention program on cardiovascular risk, self-efficacy and health promoting behaviours among patients with metabolic syndrome: Randomized controlled trial. Int J Nurs Stud. (2020) 109:103638. 10.1016/j.ijnurstu.2020.103638 32553996

[15] Rashidi A Kaistha P Whitehead L Robinson S. Factors that influence adherence to treatment plans amongst people living with cardiovascular disease: a review of published qualitative research studies. Int J Nurs Stud. (2020) 110:103727. 10.1016/j.ijnurstu.2020.103727 32823026

[16] Pender NJ Murdaugh CL Parsons MA. Health Promotion in Nursing Practice (7th Edition). New York, NY: Person Education. (2015).

[17] Baker E Fatoye F. Patient perceived impact of nurse-led self-management interventions for COPD: a systematic review of qualitative research. Int J Nurs Stud. (2019) 91:22–34. 10.1016/j.ijnurstu.2018.12.004 30669076

[18] Jones KO Lopes SS Kelly C Welsh RS Chen L Wilson M. A qualitative study on participants' experiences with a community-based mindful walking intervention and mobile device activity measurement. Complement Ther Med. (2021) 57:102640. 10.1016/j.ctim.2020.102640 33388390

[19] Torres-Vigil I Cohen MZ Million RM Bruera E. The role of empathic nursing telephone interventions with advanced cancer patients: a qualitative study. Euro J Oncol Nursing. (2021) 50:101863. 10.1016/j.ejon.2020.101863 33246247 PMC7946749

[20] Tong A Sainsbury P Craig J. Consolidated criteria for reporting qualitative research (COREQ): a 32-item checklist for interviews and focus groups. Int J Qual Health Care. (2007) 19:349–57. 10.1093/intqhc/mzm042 17872937

[21] Moser A Korstjens I. Series: Practical guidance to qualitative research. Part 3: Sampling, data collection and analysis. Euro J General Pract. (2018) 24:9–18. 10.1080/13814788.2017.1375091 29199486 PMC5774281

[22] Bryman A. Social Research Methods (4th Eds). Oxford and New York: Oxford University Press. (2012).

[23] Steckler A Linnan L. Process Evaluation for Public Health Interventions and Research. San Francisco, CA: Jossey-Bass. (2002).

[24] Graneheim UH Lundman B. Qualitative content analysis in nursing research: concepts, procedures and measures to achieve trustworthiness. Nurse Educ Today. (2004) 24:105–12. 10.1016/j.nedt.2003.10.001 14769454

[25] Lincoln YS Guba EG. Naturalistic Inquiry. Newburry Park, CA: Sage Publications (1985).

[26] Holloway ISW. Qualitative Research in Nursing and Healthcare (4th Eds). Chichester, West Sussex, U.K.; Ames, Lowa: Wiley- Blackwell. (2013).

[27] Tufford L Newman P. Bracketing in qualitative research. Qual Soc Work. (2012) 11:80–96. 10.1177/1473325010368316

[28] Wang WR Thompson DR Chair SY Twinn SF. Chinese couples' experiences during convalescence from a first heart attack: a focus group study. J Adv Nurs. (2008) 61:307–15. 10.1111/j.1365-2648.2007.04529.x 18197865

[29] Li YP Jaddoe VW Lu Q He YN Wang D Lai JQ. Exposure to the Chinese famine in early life and the risk of metabolic syndrome in adulthood. Diabetes Care. (2011) 34:1014–8. 10.2337/dc10-2039 21310886 PMC3064015

[30] Wang ZH Dong HY Xu RB Wang XJ Li YH Zou ZY. Early-life exposure to the Chinese great famine and later cardiovascular diseases. Int J Public Health. (2021) 66:603859. 10.3389/ijph.2021.603859 34744570 PMC8565276

[31] Qiu XCH Sit JWH Koo FK. The influence of Chinese culture on family caregivers of stroke survivors: a qualitative study. J Clin Nurs. (2018) 27:e309–19. 10.1111/jocn.13947 28677123

[32] Pazzagli C. Mazzeschi C. Laghezza. L/Reboldi G. P. De Feo P. (2013). Effects of a multidisciplinary lifestyle intervention for obesity on mental and physical components of quality of life: the mediatory role of depression. Psychologic Rep. 112:33–46. 10.2466/06.13.15.PR0.112.1.33-46 23654025

